# Integrating SolVES and social media analytics to quantify social value spatial patterns and driving mechanisms in mega-scale green spaces

**DOI:** 10.1371/journal.pone.0345601

**Published:** 2026-04-17

**Authors:** Hui Fan, Rongrong An, Ziyu Teng, Ying Wang

**Affiliations:** School of forestry & Landscape architecture, Anhui Agricultural University, Hefei, Anhui, China; Zhejiang Agriculture and Forestry University: Zhejiang A and F University, CHINA

## Abstract

Assessing the social values (SVs) of mega-scale green spaces (MSGs) in regenerated brownfields remains challenging due to spatial heterogeneity and perceptual dynamics. This study developed a dual-modal framework integrating the Social Values for Ecosystem Services (SolVES) model and social media analytics (1,086 reviews) to assess SVs in Luogang Park (1,270 ha), Hefei. Results revealed: (1) spatial polarization of SVs, with aesthetic and recreational hot spots clustering in cultural landmarks (mean value index, M-VI = 10), while biodiversity cold spots (M-VI = 6) were dispersed in ecological zones; (2) key drivers included proximity to roads (20–50 m buffer, contribution = 32.7%) and density of service facilities (p < 0.01); and (3) ecological values (e.g., life-sustaining functions) received 58% less public attention than aesthetic values, as identified through semantic analysis. Based on these findings, spatial optimization strategies-such as cultural-recreational corridors and nature education zones-along with a diversified management mechanism were proposed. The framework advances ecosystem services (ES) assessment by reconciling spatial quantification (AUC > 0.8) and semantic perception, offering a transferable tool for equitable and multifunctional green space planning.

## 1. Introduction

As global urbanization transitions from incremental expansion to stock optimization, large green spaces—commonly termed mega-scale green spaces (MSGs)—are gaining prominence. Once serving primarily as ecological buffers on the urban periphery, these spaces now evolve into multifunctional areas that integrate production, residential, and ecological roles at the city core [1–3]. In addition to their conventional ecological functions of preserving biodiversity and mitigating urban heat islands [4,5], MSGs have evolved into essential infrastructure that bolsters urban resilience and fosters social equity [6]. MSGs are defined as contiguous areas exceeding 50 hectares (500,000 m^2^); studies have shown that patches of this size can significantly enhance ecological connectivity, carbon sequestration, and urban cooling [7,8]. Urban mega-green spaces deliver essential ecosystem services—biodiversity conservation, climate regulation, and hydrological management and provide multifaceted socio-ecological services such as public health enhancement, cultural heritage preservation, and social capital development [9].

However, despite growing recognition of their multifunctionality, existing studies often overlook the spatial heterogeneity of social values associated with MSGs, and there remains a lack of integrative frameworks capable of capturing both ecological and perceptual dimensions. This gap is particularly pronounced in the context of brownfield regeneration, where unique socio-environmental legacies—such as collective memory, remediation awareness, and identity reconstruction—impart multidimensional social values that current ecosystem service frameworks seldom quantify [10,11]. Methodologically, a significant disconnect persists. While the SolVES model has been applied to small- or medium-scale parks and natural reserves [12,13], its application in large, regenerated brownfields is limited. Existing studies utilizing SolVES primarily focus on ecological or biophysical aspects, overlooking the perceptual and socio-cultural dimensions in revitalized environments [14]. Conversely, research focused on perception using social media data is predominantly descriptive and lacks spatial coupling and explanatory capacity [15]. Consequently, a conceptual framework and methodological approach for integrating spatial modeling and perception analysis in large-scale regenerated parks are notably deficient [16,17].

A comprehensive literature review exposes significant theoretical and methodological shortcomings in assessing the social value of mega-scale green spaces (MSGs), especially those revitalized from brownfield sites. The current ecosystem services (ES) evaluation framework, based on the four-dimensional categorization system of the Millennium Ecosystem Assessment (MA, 2005) [18,19], predominantly offers static and descriptive analyses of social values, constraining its ability to measure intangible aspects such as aesthetic, spiritual, and forward-looking advantages [20,21]. Conventional economic models like the Contingent Valuation Method (CVM) tend to oversimplify these values by assuming linear relationships and disregarding spatial heterogeneity, which fundamentally influences public perception and experiences [14,22]. This lack of attention to spatial and perceptual variability has led to a persistent mismatch between planning strategies and the actual socio-cultural needs of the public [23].

Although social media analytics have recently emerged as a novel means of capturing public sentiment, their capacity to monitor evolving social values within complex contexts—such as brownfield regeneration—remains constrained by overreliance on single data sources and limited multimodal integration [24,25]. Current assessment models often fail to consider the socio-environmental histories that define brownfield landscapes. Factors such as historical memory—which reflects community identity shaped by past land uses—and environmental justice—which pertains to perceptions of fair remediation and benefit distribution—are crucial in shaping social values. However, they are frequently overlooked in existing frameworks [26,27]. Methodologically, a significant gap persists: while the SolVES model has been widely applied to ecological evaluations in small- to medium-scale natural areas, it largely neglects perceptual dimensions and human–environment interactions in regenerated urban landscapes [28,29]. Conversely, perception-based studies utilizing social media data often lack spatial integration and explanatory depth, hindering the identification of spatial–cognitive mechanisms underpinning social value formation [30–32]. Consequently, there remains a critical absence of an integrated framework that synthesizes spatial modeling and perceptual analysis to holistically capture the socio-spatial dynamics of social values in large-scale regenerated parks [33,34].

This study aims to address these gaps by constructing an integrated analytical framework that combines the SolVES model with social media analytics to quantify the spatial patterns and driving mechanisms of social values in MSGs. The following sections review the functional transformation of MSGs, the evolution of social value concepts, the role of public perception, and the methodological innovations that inform this perception-driven research framework [35,36]. This research proposes an innovative dual-modal approach, designated as “spatial quantification–semantic perception,” to evaluate the social value of Luogang Park—a representative mega-scale green spaces (MSGs) developed from a reclaimed brownfield site. By integrating the SolVES model with advanced social media analytics—including LDA topic modeling, sentiment analysis, and social network analysis—this research transcends the limitations of single-method approaches prevalent in cultural ecosystem service (CES) assessment [37,38]. The framework’s predictive reliability provides a foundation for quantifying spatial variations in social values and identifying the driving effects of key environmental variables [39].

The concept of “semantic perception” involves the structured extraction and spatial integration of public perceptions, attention patterns, and emotional sentiments from unstructured social media data. This goes beyond basic descriptive analytics by revealing the socio-cultural influences, such as ingrained memories and perceptions of environmental justice, that influence the spatial distribution of social value [35]. This integrated approach fills a critical gap in the literature on Cultural Ecosystem Services (CES), which has traditionally treated spatial quantification and perceptual analysis as separate domains [40]. While our research findings align with previous studies on optimizing large green spaces through cultural-entertainment corridors and nature education zones [41], our study provide novel insights into the functioning of these elements within a post-industrial context. Specifically, we examine how they reconcile historical heritage with contemporary recreational needs. In contrast to conventional Ecosystem Services (ES) assessments that often neglect the unique socio-environmental histories of brownfield sites [42], our framework specifically captures how historical memory and concerns about environmental justice influence social values in the revitalized MSG area. This marks a significant theoretical advancement by broadening the CES assessment framework to consider the intricate interplay between past land use, awareness of remediation efforts, and the evolving identity of the community. The dynamic “spatial function connection – geographical element matching” mechanism further provides an original contribution to spatial optimization theory, offering a transferable approach for linking high- and low – value areas while addressing equity considerations in urban regeneration contexts [32,43].

This study contributes methodologically and theoretically to the field of CES assessment by integrating historical memory and environmental justice into an analytical framework. It provides planners and policymakers with practical insights for designing more equitable, socially responsive mega-scale green spaces. The research highlights that successful brownfield regeneration necessitates not only ecological enhancement but also deliberate engagement with community heritage and perceptual dimensions, presenting a replicable model for sustainable urban development in post-industrial landscapes [35].

## 2. Research methods

### 2.1. Overview of the study area

**Fig 1:**
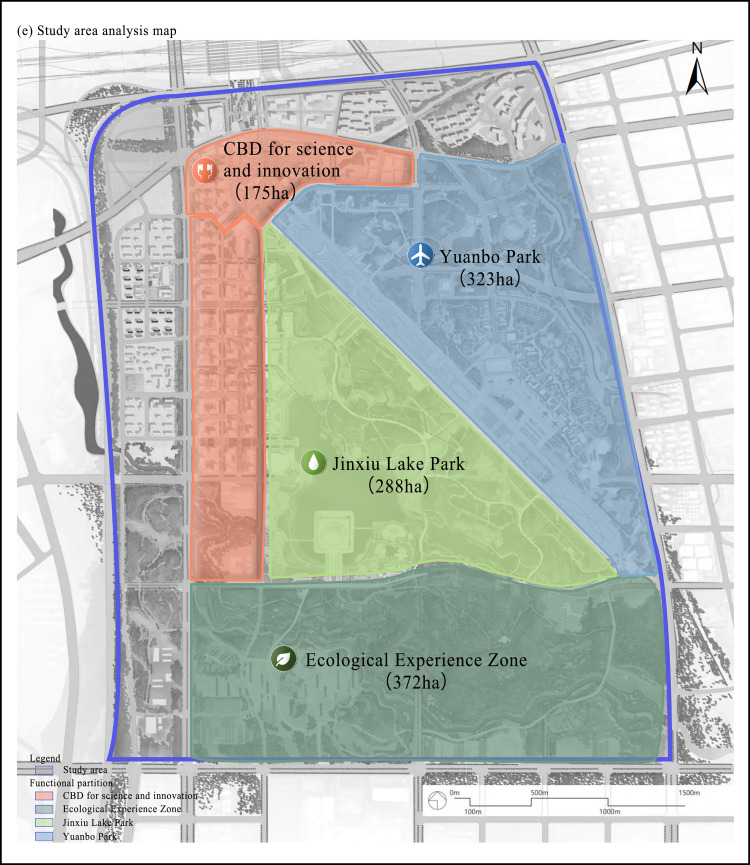
Regional Location Map (The map was simplified and redrawn by the authors based on fieldsurveys and coordinates; no copyrighted base maps were used).

The study area, Luo-gang Park, is located in the Baohe District of Hefei, Anhui Province, China (31°46’ to 31°49’ N, 117°16’ to 117°19’ E). Originally the site of the city’s international airport, the area has been transformed into one of the largest comprehensive urban parks in the world, spanning 12.7 km2. Its spatial layout is characterized by a central axis extending north-to-south, which integrates ecological restoration zones with diverse recreational facilities, making it an ideal site for examining the driving mechanisms of perceived social values. It is situated on the former Luogang Airport site, which was established in 1953 and decommissioned in 2013 (Fig 1). As a representative example of brownfield regeneration within urban stock renewal, its transformation encompasses ecological restoration, functional enhancement, and cultural revitalization. The site’s profound industrial heritage and collective place memory critically mediate public perceptions of environmental remediation and landscape change, thereby shaping place attachment and social identity. Nonetheless, current social value assessment frameworks often neglect the complex role of historical continuity and place memory in influencing public valuation [44,45]. Addressing this deficiency is essential to inform context-sensitive regeneration strategies that integrate heritage preservation with ecological and social benefits, ultimately advancing sustainable urban development.

The park comprises the Garden Expo Park, Jinxiu Lake, Sci-Tech Innovation CBD, and an ecological experience area. It integrates diverse functions, including the 14th China International Garden Expo exhibition gardens, digital economy, sci-tech innovation finance, scientific exchanges, high-end commerce, farmland, and urban ecological wetlands. Since opening, the park has hosted over 800 events, drawing substantial tourist numbers. By October 2024, visitor numbers surpassed 15 million. During the 2024 “May Day Holiday” the park recorded a peak single-day attendance of 360,000, with 46% of visitors from outside the province, marking a new record for daily attendance.

### 2.2. A dual-modal framework: Integrating spatial quantification and semantic perception

**Fig 2:**
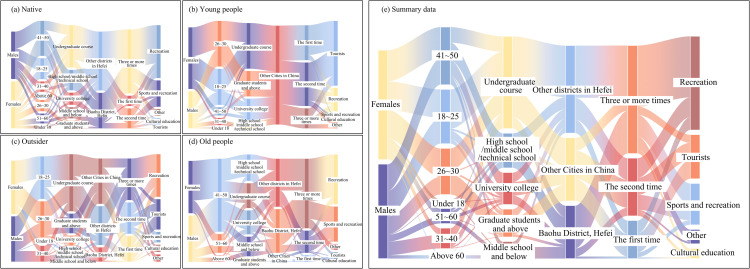
Research framework.

The research constructs a dual-modal framework that integrates the Social Values for Ecosystem Services (SolVES) model with social media analytics to comprehensively assess the social values (SVs) of ecosystem services in Luogang Park and uncover their underlying drivers [46]. This framework is designed to synergize data from field surveys and user-generated textual content, enabling a holistic analysis that bridges spatial modeling with public perception [47]. The overall workflow of this framework is illustrated in Fig 2.

#### 2.2.1. Theoretical foundation.

The methodological design is grounded in the Social-Ecological Systems (SES) framework, which conceptualizes ecosystem service values as dynamic outcomes of continuous interactions between ecological conditions and human perceptions [48]. This aligns with recent advances in perception-based ecosystem service studies that advocate for the recognition of co-produced and relational values [49]. Based on this foundation, our dual-modal framework operationalizes a “spatial quantification—semantic perception” approach. This approach integrates two components: (1) spatial quantification via the SolVES model to capture the environmental determinants and spatial distribution patterns of social values, and (2) semantic perception analysis of social media text data to identify the subjective, culturally embedded, and emotional dimensions of public experience [48,50].

#### 2.2.2. SolVES model: Spatial quantification of social value.

The Social Values for Ecosystem Services (SolVES, v4.0) model was adopted to quantify the spatial distribution of social values associated with ecosystem services within Luogang Park. This model enables the integration of social survey–derived value points with multiple environmental variables to simulate the spatial heterogeneity of perceived values.

A suite of spatial environmental variables—including distance to roads (DTR), distance to water bodies (DTW), land use/land cover (LULC), and the density of service facilities—was incorporated into the SolVES environment to examine how ecological and accessibility factors influence social value distribution. The Maximum Entropy (MaxEnt) algorithm was applied to evaluate the relative contributions and response curves of these variables, providing insights into the spatial determinants of perceived social value [51].

The model performance was validated using receiver operating characteristic (ROC) curves and the area under the curve (AUC), both exceeding 0.8, confirming high predictive accuracy and robustness. These results establish a reliable quantitative basis for linking environmental characteristics with social value perception and set the foundation for subsequent semantic perception analysis.

#### 2.2.3. Social media analysis: Semantic perception and sentiment mapping.

This study employed a text-mining framework integrating natural language processing (NLP) and social network analysis to capture the dimension of social values defined as “semantic perception” [52]. This concept denotes the process of extracting and interpreting the underlying meanings, attitudes, and cultural values that the public associates with a landscape from unstructured textual data. It transcends basic sentiment analysis by revealing how socio-cultural constructs, including historical memory and perceptions of environmental justice, shape the subjective experience of ecosystem services. User-generated content from major Chinese social media platforms was collected using the Scrapy crawler framework and processed through a standardized cleaning pipeline that included deduplication, tokenization, and stop-word removal [53,54].

The preprocessed corpus was subsequently analyzed using multiple complementary techniques. Word frequency statistics were first applied to extract salient terms that reflect public attention. Latent Dirichlet Allocation (LDA) topic modeling was then employed to identify latent social value themes and their correspondence with cultural ecosystem service dimensions. Keyword co-occurrence networks were constructed to visualize thematic interrelations and the relative salience of value categories, revealing public perception structures [55]. Finally, sentiment analysis using a lexicon-based approach quantified emotional polarity, thereby linking affective expression with specific landscape features and spatial contexts. This multi-stage semantic analysis enables a systematic interpretation of public discourse, uncovering both explicit and implicit dimensions of social value perception that complement the spatial quantification results derived from the SolVES model.

#### 2.2.4. Integration and cross-validation.

The dual-modal research framework accurately illustrates the spatial distribution of the social value of ecosystem services in Luogang Park and general public attitudes. By integrating spatial environmental drivers with socio-cultural regulatory factors, it systematically examines the comprehensive mechanisms shaping social value [56,57]. This framework offers a scientific foundation for spatial optimization and dynamic management of Luogang Park and serves as a methodological model for studying other extensive urban green spaces.

### 2.3. Data sources (questionnaires, social media, geospatial)

#### 2.3.1. Social survey data.

Public visitors’ perceptions were gathered through on-site questionnaires administered via a stratified random sampling method across various park zones and time periods, including weekdays, weekends, and holidays, from September to December 2024. This design ensured both temporal and spatial representation, thereby minimizing potential biases. Out of 327 distributed questionnaires, 300 were deemed valid, resulting in a validity rate of 91.74%. The sample demonstrated diversity and balance: gender (53.67% female, 46.33% male), age (spanning 18–60 years), and residence (69% Hefei residents vs. 31% non-resident visitors). Visiting motivations included tourism (42.7%), leisure & entertainment (33.1%), and sports & fitness (24.2%), aligning with the park’s multifunctional purpose. Cross-tabulation analyses confirmed no statistically significant differences (p > 0.05) between key demographic variables (age, gender, residence) and spatial-use frequency, underscoring that the sample effectively captures the behavioral diversity of major user groups and strengthens the generalizability of our findings.

**Fig 3:**
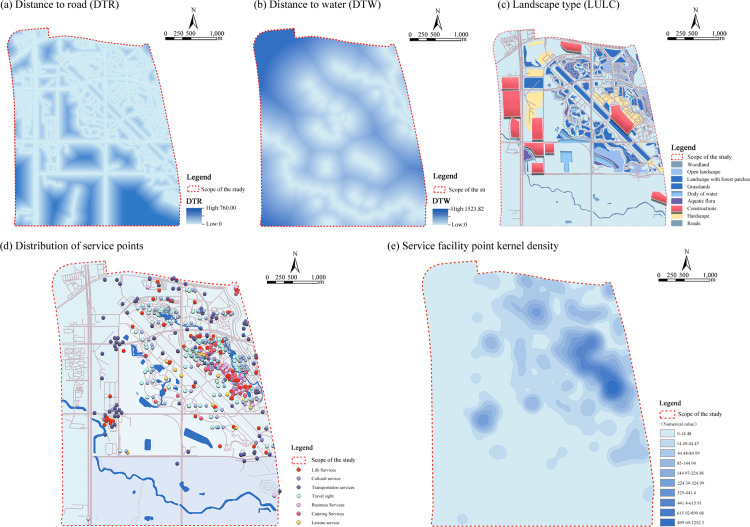
Differences in the assessment of social values between resident and non-resident visitors.

In view of the diversity of visitor subjects in social media data, the samples were categorized into youth and middle-aged and old-aged groups based on age characteristics, and into resident and non-resident visitor groups according to the characteristics of the purpose of use. Six of the eleven categories of social values were selected to explore the differences in the assessment of social values of Luogang Park by different public groups (Fig 3). The questionnaire comprised three sections: (1) Respondents’ basic information; (2) Characteristics of public engagement; (3) Ratings of social value and identification of 1–4 locations within the park that best represent these values.

Social value points were designated by visitors identifying 1–4 scenic spots epitomizing each value type. Informed by Sherrouse’s typology and the park’s “ecology first” ethos, 11 social values (e.g., aesthetics, biodiversity, recreation) were initially selected. For integration with social media data, these were refined to five core dimensions—aesthetic, economic, therapeutic, recreational, and life-sustaining. This refinement was based on their empirical relevance to park use, frequency and clarity in textual data, and semantic distinctiveness. The resulting taxonomy aligns SolVES and social media classifications while preserving key socio-ecological values.

#### 2.3.2. Acquisition and processing of environmental variables.

Geographical environmental variables are critical for assessing the social value of ecosystem services. Luogang Park in Hefei features predominantly flat terrain with minimal variation in elevation and slope, which limits their influence on the spatial distribution of ecosystem service social values. Digital Elevation Model (DEM) analysis has shown that the standard deviation of elevation within the study area is less than 2 meters, and average slope values remain below 3°, confirming negligible topographic relief. Consequently, elevation and slope were excluded from the set of environmental variables influencing social value patterns, as their impact was minimal compared to other factors such as proximity to water bodies or accessibility. This quantification supported the assumption that terrain variability did not significantly affect ecosystem service valuation within Luogang Park.

Consequently, this study selects distance to roads (DTR) and distance to water bodies (DTW) as spatial proximity variables. These metrics directly capture spatial relationships between key elements and are essential for understanding public accessibility and preferences regarding different park zones.

**Fig 4:**
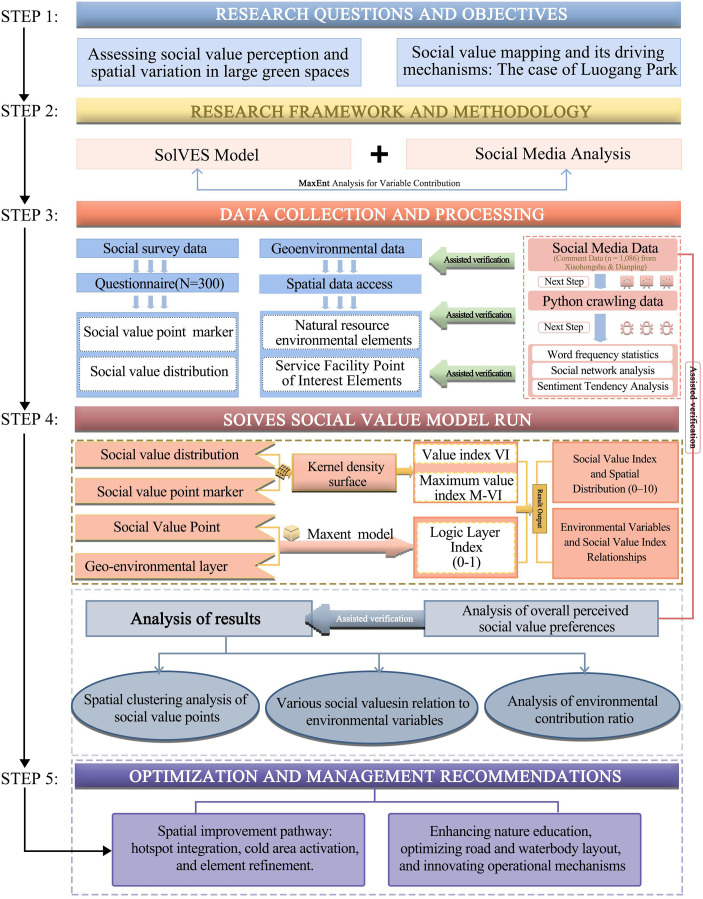
Relationship between various types of social values and natural factor conditions.

To capture the park’s spatial characteristics, land use and land cover (LULC) are included as environmental variables to reflect landscape functions and land-use patterns. Recognizing the link between urban parks and human well-being, we also consider the point density of seven service facility categories-daily, cultural, transportation, tourist attractions, commercial, catering, and recreational services-as additional environmental variables. These service facility densities reflect the spatial distribution of park functions and play a direct role in shaping public perceptions and utilization of social values (see Table 1, Fig 4).

**Table 1:** Types and descriptions of social values and some representative high frequency words.

Ecosystem service categories	Social value categories	Value Description	Some representative high-frequency words
Social service	aesthetic value	The park’s scenic and pleasant setting and the strong visual appeal of the park’s natural landscapes are important to me.	Featured, Architecture, Good-looking, Garden, Style, Moon Festival, Garden Expo, Hundred Gardens, Red Walls
	spiritual value	The garden can make me feel the beauty of nature and purify my mind, which is important to me.	–
	cultural value	The park has given me access to wisdom, knowledge, culture, and the way of life of my ancestors, which is important to me.	–
	future value	It is important to me that parks provide opportunities for the next generation to learn about and experience urban parks and green spaces.	–
	intrinsic value	The park itself has value, whether or not it is displayed	–
	entertainment value	It is important to me that parks have ample recreational space with amenities and programs that are enjoyable to me.	Hits, photos, food, entertainment, eating and drinking, helicopters, kite flying, garden towns
	Recreational value	The park provides me with the recreational space and facilities I need to be physically and mentally healthy. It’s a place to exercise, soothe my body and mind, and release stress, which is important to me.	Lawn, Kids, Facilities, Recreation, Track, Walk, Playground
	scientific research value	The park provides a place for scientific investigation and experimentation, which is important to me.	–
economic service	economic value	Parks provide opportunities to visit, drive tourism, and are important to me.	Hotels, light shows, music festivals, garden towns, tourism, specialty, helicopters
ecological service	Sustainable value for life	Parks are important to me because they produce matter, purify water, purify the atmosphere, and preserve soil and water.	Landscape, relaxation, excursion, ecology, cozy, air, nature, trekking
	Biodiversity values	The park is rich in flora and fauna and other things that are important to me.	–

**Fig 5:**
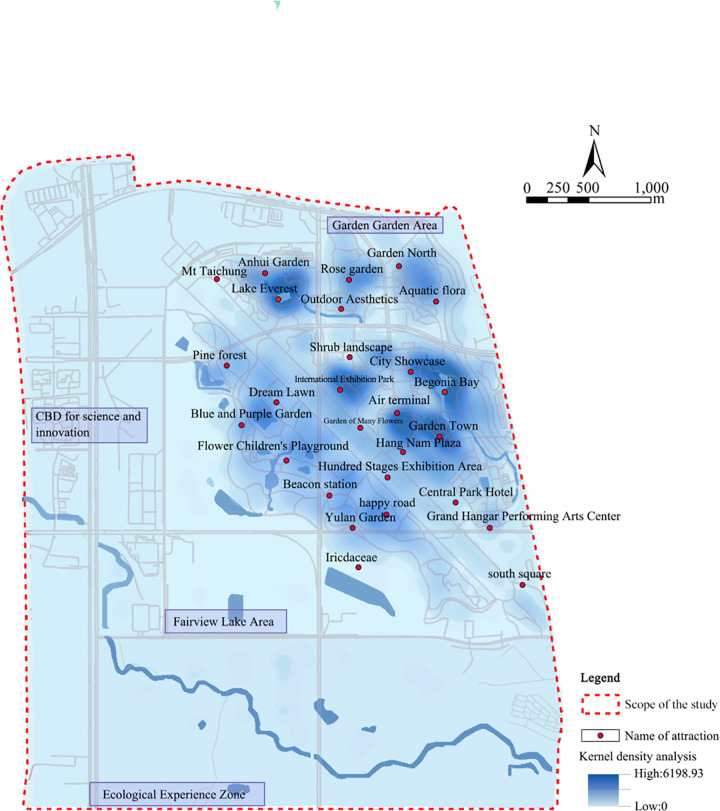
Luo gang Park Environmental Elements Layer. (The map was simplified and redrawn by the authors based on fieldsurveys and coordinates; no copyrighted base maps were used.).

In summary, the study has selected ten geographic environmental variables-three natural resource factors (DTR, DTW, and LULC) and seven service-facility point-density factors. These variables have been integrated into a comprehensive evaluation model to quantify the spatial distribution characteristics and influencing mechanisms of the social value of ecosystem services in Luogang Park. The analysis of service-facility point densities and distributions helped identify hot spots of human activity and provided essential support for understanding the intensity of public perception and variations in spatial value. Consequently, service-facility density serves as a key linkage between spatial structure and perceived social value (see Table 2, Fig 5).

**Table 2:** Geospatial data description.

Geospatial data		Data description	Data sources
Natural resources environment	DTW	Distance to body of water DTW	Euclidean distance tool in ArcGIS.
	DTR	Distance from roadway DTR	Obtained by using the Euclidean Distance tool in ArcGIS.
	LULC	Landscape space type	--
Service Facilities Points of Interest	Life Services	Density of facilities such as public restrooms, ticket counters, luggage storage, visitor service centers, greenway stations, hotel accommodations, etc.	Gaode Map (Chinese version of GPS)
	Cultural Services	Density of facilities such as museums, places for commemorating the history of urban development and ecological culture, bookstores, etc.	Gaode Map (Chinese version of GPS)
	Transportation	Density of facilities such as parking lots, bus stops, sightseeing stations, and tourist stops	Gaode Map (Chinese version of GPS)
	Tourist Attractions	Density of facilities such as attractions, city gardens, amusement parks, zoos, and scenic rides	Gaode Map (Chinese version of GPS)
	Commercial Services	Density of facilities such as convenience stores, supermarkets, stores, etc.	Gaode Map (Chinese version of GPS)
	Catering Services	Density of facilities such as restaurants, cafes, snack stores, cafeterias, cake and dessert stores, bars, etc.	Gaode Map (Chinese version of GPS)
	Leisure Services	Density of facilities such as bed and breakfasts, sports and fitness facilities, etc.	Gaode Map (Chinese version of GPS)

#### 2.3.3. Social media data.

While Weibo and Douyin also generate substantial park-related content, both were excluded due to structural limitations: Weibo is news-and opinion-oriented with weak geographic binding, offering limited place-specific experiential description, while Douyin’s content is primarily entertainment-driven and algorithmically distributed, with textual components confined to fragmented subtitles unsuitable for systematic semantic analysis. By contrast, Xiaohongshu and Dianping offer strong place-specificity and complementary perceptual coverage—the former capturing affective and experiential dimensions through detailed lifestyle narratives, the latter providing structured service evaluations across a broader demographic. Together, they address both perceptual and functional dimensions of social value aligned with our analytical framework. To mitigate residual platform-specific biases, data normalization and frequency-weighted co-occurrence network analysis were applied. The spatial consistency between social media-derived value patterns and SolVES-modeled hot spots further supports the adequacy of this combined dataset. Their integration provides a more holistic understanding of both perceptual and behavioral dimensions of social value. To ensure data quality and validity, the collected raw data underwent meticulous cleaning. This process involved removing duplicate and irrelevant content (e.g., commercial advertisements, content-less check-in posts, and inquiries about lost items), as well as standardizing non-standard expressions (e.g., replacing “yyds” with “excellent” and contextualizing “Clock in” as “visit” or “check-in”).

After the above processing, a total of 1086 valid comments were finally obtained. Subsequently, a word frequency analysis was performed on the segmented text data, and the top 80 high-frequency keywords were extracted from the segmented word frequency table to identify the public’s focus of attention on Luogang Park. We acknowledge potential platform-specific biases (e.g., Xiaohongshu’s overemphasis on visual appeal vs. Dianping’s consumption-oriented focus). To mitigate these, we applied data normalization and frequency-weighted co-occurrence network analysis to balance the influence of different perception categories.

The text data were segmented using the Jieba library in Python, and the top 80 high-frequency keywords—determined by the elbow method—were extracted for analysis. Based on the three major categories and eleven sub-categories of social values in our ecosystem services framework, these keywords were systematically classified into five core value dimensions for subsequent analysis. To mitigate potential platform-specific biases (e.g., Xiaohongshu’s aesthetic emphasis vs. Dianping’s consumption focus), we applied data normalization and frequency-weighted co-occurrence network analysis. The observed spatial consistency between social media-derived perception patterns and SolVES-modeled value distributions further supports the complementarity of the selected platforms in capturing both perceptual and functional dimensions of social value. The results are shown in Table 1 (Table 1).

#### 2.3.4. Technological route.

See Fig 2.

## 3. Results

### 3.1. Assessment of social values and their spatial distribution

#### 3.1.1. Kernel density analysis of social value points.

**Fig 6:**
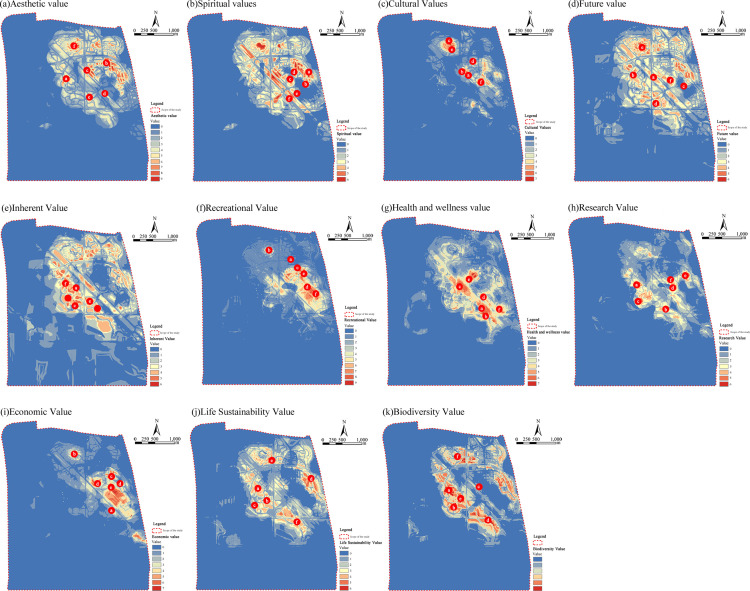
Density distribution of social value point kernel in Luogang Park, Hefei City (The map was simplified and redrawn by the authors based on fieldsurveys and coordinates; no copyrighted base maps were used).

A kernel density analysis was conducted on 4,043 social-value points provided by 300 respondents to identify “ hot spots ” (high – value areas) and “cold spots” (low-value areas) within Luogang Park, Hefei. High-density clusters of aesthetic and recreational values were primarily located in the Urban Exhibition Garden, Yuanbo Town, and Anhui Garden sub-areas, which are characterized by diverse landscapes and well-equipped service infrastructure. In contrast, the Jinxiuhu sub-area presented a lower density of hot spots, with social value points more frequently associated with health and wellness activities. This distribution aligns with the ecological layout of the area and the presence of natural, restorative environments (Fig 6). Across the park, aesthetic and recreational values were more commonly concentrated in intensively developed functional zones, while health-related values were more prevalent in zones with a stronger ecological orientation.

### 3.2. Spatial clustering analysis of social value points

Spatial cluster analysis was applied to the social-value points collected in Luogang Park, Hefei. The results indicated that all value categories exhibited statistically significant spatial agglomeration (R < 1; Z-value < 0), confirming a clustered rather than random distribution (Table 3). The ranking of maximum-value indices (M-VI) was as follows: aesthetic = entertainment (10)> cultural = health-care = economic (8)> spiritual (7)> future = intrinsic = scientific-research (6)> life-sustainability = biodiversity (6). Among these, aesthetic and entertainment values exhibited the highest degree of spatial concentration, followed by cultural and economic values.

**Table 3:** Maximum value index and spatial clustering analysis of various values in Luogang Park.

Type of social value	Total number of social value points N/each	Maximum value index M-VI	Nearest neighbor ratio R value	Standard deviation Z value
Aesthetic value	387	10	0.558469	−16.6168
Spiritual values	374	7	0.60404	−14.6494
Cultural Values	400	8	0.420568	−22.1699
Future value	373	6	0.623179	−13.9226
Inherent Value	365	6	0.624381	−13.7285
Recreational Value	398	10	0.530855	−17.9052
Health and wellness value	376	8	0.609806	−14.4746
Research Value	312	6	0.549475	−15.2239
Economic Value	370	8	0.473083	−19.3898
Life Sustainability Value	348	6	0.557421	−15.7947
Biodiversity Value	340	6	0.51097	−17.2507

R-values and Z-values indicate the results of spatial clustering of the social value of ecosystem services, with R < 1 indicating clustering, R = 1 indicating randomization, and R > 1 indicating dispersion.

**Fig 7:**
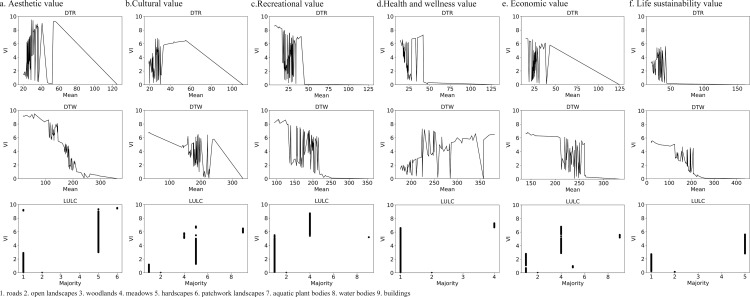
Spatial distribution of various types of social values in Luogang Park(The map was simplified and redrawn by the authors based on fieldsurveys and coordinates; no copyrighted base maps were used).

Aesthetic and entertainment hot spots were primarily located in the core landscape belt encompassing the Anhui Garden, Urban Exhibition Garden, and Hundred-Flower Garden (kernel density ≥ 0.85 km^−2^), as well as along the entertainment corridor connecting Luogang Town and the Urban Exhibition Garden (kernel density ≥ 0.78 km^−2^). These zones are characterized by diverse landscape compositions and extensive recreational facilities. These zones are characterized by diverse landscape compositions and extensive recreational facilities. Cultural-value hot spots were concentrated around iconic features such as the former airport terminal and Huizhou-style architectural complexes, with a high Moran’s I value (0.67, p < 0.01), corresponding to the spatial imprint of historical and cultural landmarks. High-density health-care values were found along the slow-traffic waterfront in the Jinxiu Lake area, where vegetation cover was high (NDVI ≥ 0.7), and the environment supported restorative functions. Economic value was spatially clustered within the Garden Expo business town, where commercial infrastructure was concentrated, and perceived economic benefits were more pronounced (Table 3; Fig 7).

Latent values-including intrinsic, future, biodiversity, and life-sustaining values (M-VI = 6) – exhibited a dispersed spatial pattern throughout Luogang Park, with only limited clustering observed in the ecological conservation zone surrounding Jinxiuhu Lake. These values were primarily associated with aquatic and low-disturbance habitats. Compared to more prominent categories such as aesthetics and recreation, their overall perceived intensity was substantially lower. This disparity corresponded with spatial differences in visitor activity intensity, as areas with high levels of human presence provided greater exposure to visually or functionally explicit values, while low-visitation zones offered fewer perceptual cues for latent value recognition.

The Urban Exhibition Garden, one of the park’s most intensively developed zones, accounted for approximately 62% of total visitor traffic and received the highest concentration of aesthetic and recreational social-value points. In contrast, ecologically oriented areas recorded a hot-spot absence rate exceeding 75% for latent value categories, despite the presence of high-quality natural features. This pattern aligned with a differentiated “functional zoning - perception response” in which distinct spatial functions were associated with varying degrees of public attention to different social value dimensions.

Across the entire park, the spatial distribution of social values demonstrated marked heterogeneity (Fig 4). Aesthetic and recreational value clusters were concentrated in core landscape areas and along leisure corridors. Cultural values aggregated near heritage landmarks such as the former terminal and Huizhou-style buildings. High-density health and wellness values were located along riparian greenways, while economic value was centered in commercial and service-oriented zones. In contrast, latent values remained scattered and showed minimal spatial aggregation, even in conservation-dominant areas. The overall distribution captured prevailing public preferences and underscored the structuring influence of spatial function on the perception and localization of ecosystem service values.

### 3.3. Relationship between social values and environmental variables

To examine the relationship between environmental variables and the social value indices (SVIs) of ecosystem services, the Social Values for Ecosystem Services (SolVES) model was employed. Drawing on an integrated assessment of both the typology and distribution of social values in Luogang Park, six representative categories were selected for modeling: aesthetic, cultural, recreational, health and well-being, economic, and life-sustaining values. Response curves were generated to depict the variation in SVIs across gradients of key environmental factors. These variables included elevation, slope, distance to roads, distance to water bodies, land use type, and normalized difference vegetation index (NDVI). The resulting curves characterize the sensitivity of each value category to environmental conditions and provide a basis for identifying spatial thresholds associated with high or low perceived social value.

Building on the nearest neighbor statistics (Table 3), social value agglomeration patterns can be differentiated into three structural types based on M-VI rankings and kernel density distributions (Fig 6).

High-density concentration (M-VI = 10): Aesthetic and recreational values showed the strongest clustering, with hot spots concentrated in the Urban Exhibition Garden, Yuanbo Town, and Anhui Garden—sub-areas characterized by intensive landscape design and well-developed service infrastructure that reinforce engagement with visually prominent values.

Moderate agglomeration (M-VI = 7–8): Cultural, health-care, economic, and spiritual values formed moderately clustered patterns across activity-oriented zones, reflecting the presence of specific attractions-cultural heritage elements, waterfront promenades, and commercial nodes—that draw targeted visitor groups rather than broad-based concentration.

Dispersed distribution (M-VI = 6): Biodiversity, life-sustaining, intrinsic, future, and scientific-research values showed diffuse distributions across the Jinxiuhu wetland conservation area. Despite its ecological significance, restricted access and limited interpretive infrastructure reduce the zone’s perceived social value.

This three-tier structure aligns with the park’s functional zoning, indicating that social value agglomeration is mediated not only by ecological endowment but by accessibility, design intensity, and service provision.

#### 3.3.1. Relationships between biophysical factors and perceived social values.

The Social Values for Ecosystem Services (SolVES) model was applied to examine how environmental factors influenced the perceived social values of ecosystem services in Luogang Park. Among the key variables, distance to roads (DTR), distance to water bodies (DTW), and land-use/land-cover (LULC) exerted notable effects on social value indices. Within a 20–50 m buffer from roads, the indices exhibited pronounced variation, corresponding with zones of intensive landscape design and higher accessibility. Between 100 and 300 m from water bodies, social value indices fluctuated, reaching peak levels near 175 m, where proximity to aquatic features coincided with favorable visual and environmental conditions. In terms of land cover, areas characterized by dense road infrastructure, grasslands, and hardscaped recreational zones recorded relatively higher social value scores across multiple categories. These patterns align with the spatial characteristics of high-use areas and indicate that both accessibility and functional spatial configuration influence the spatial distribution of social values in the park (Fig 4).

#### 3.3.2. Relationships between social values and Points of Interest (POI) of service facilities.

The analysis identified a strong relationship between the density of service-facility points of interest (POIs) and the spatial distribution of perceived social values in Luogang Park. Aesthetic, cultural, recreational, and economic values showed positive correlations with the density of commercial services, daily-life services, tourist attractions, and recreational facilities. Higher concentrations of these POI types corresponded with elevated social value indices in their respective locations. Health and well-being values also exhibited positive associations with the density of tourist attractions, daily-life services, and recreational facilities, but no significant correlation was observed with cultural or transportation services. This pattern aligned with usage preferences for restorative functions, where environmental quality played a central role.

**Fig 8:**
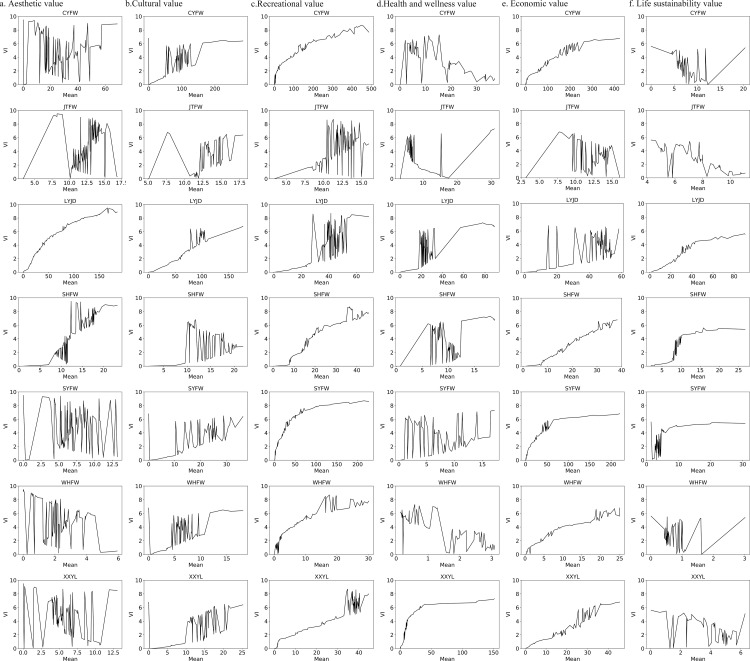
Relationship between various types of social values and the density of service facility points.

In contrast, life-sustaining values were negatively correlated with the density of catering, transportation, cultural, and recreational facilities. These values reached higher levels in areas with reduced human activity and infrastructure density, particularly in zones characterized by minimal anthropogenic disturbance. The spatial distribution indicated that different POI categories exerted differentiated influences on various dimensions of social value (Fig 8).

#### 3.3.3. Contribution analysis of environmental variables.

**Fig 9:**
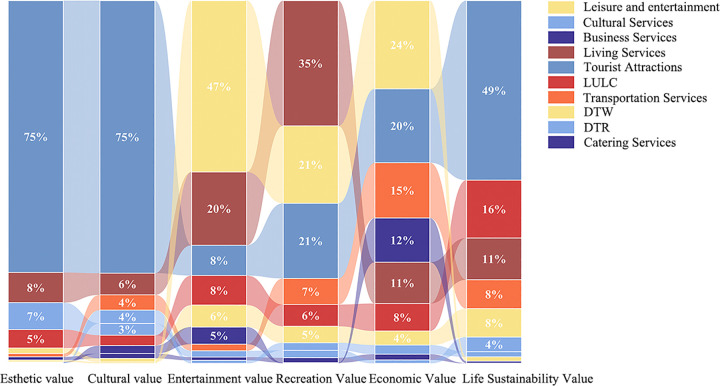
Percentage stacking of the contribution of geo-environmental elements to the distribution of social value types.

The MaxEnt model results identified distance to roads (DTR), distance to water bodies (DTW), and land-use/land-cover (LULC) as key environmental variables influencing the spatial distribution of social values in Luogang Park. DTR and LULC exhibited relatively higher contributions to aesthetic and cultural values, while DTW and LULC were more strongly associated with the distribution of recreational, health and well-being, economic, and life-sustaining values. Among anthropogenic variables, the density of tourist attractions and daily-life service facilities accounted for substantial contributions across all social value categories, indicating their importance as environmental predictors. Furthermore, commercial and recreational service facility densities made notable contributions to the spatial patterns of recreational, health and well-being, and economic values. These results demonstrate that tourism infrastructure and landscape composition serve as major drivers shaping the spatial variation of perceived social values (Fig 9).

Based on the combined application of the Social Values for Ecosystem Services (SolVES) model and the Maximum Entropy (MaxEnt) model, the analysis quantified the effects of both natural environmental features and anthropogenic service-facility distributions on ecosystem service perceptions. Proximity to roads and water bodies, along with land-use characteristics, was closely associated with multiple dimensions of perceived social value. Additionally, the density of service-related POIs significantly influenced the spatial distribution of aesthetic, cultural, recreational, health and well-being, and economic values. Together, these findings provide a data-driven foundation for optimizing spatial configurations and management strategies in large urban parks, with the aim of enhancing public access to, and awareness of, ecosystem service values.

### 3.4. AUC model validation and data comparison

To evaluate the reliability of the Social Values for Ecosystem Services (SolVES) model in assessing perceived social values in Luogang Park, the Maximum Entropy (MaxEnt) model was applied as a complementary validation tool. Model performance was assessed using the Area Under the Receiver Operating Characteristic Curve (AUC), where values range from 0 to 1, with higher values indicating stronger predictive capability. The AUC results for all categories of social values exceeded 0.8 (Table 4), demonstrating a high level of accuracy and stability in the spatial predictions generated by the SolVES model. These results confirm the suitability of the SolVES model for analyzing spatial perceptions of ecosystem services and support its application in future studies and urban park planning practices.

**Table 4:** AUC values for each type of social value in Luogang Park.

Type of value	Training AUC	Test AUC
Aesthetic value	0.9381	0.9324
Spiritual values	0.9348	0.92
Cultural Values	0.968	0.9639
Future value	0.9069	0.8596
Inherent Value	0.8848	0.8575
Recreational Value	0.9534	0.934
Health and wellness Value	0.9397	0.9144
Research Value	0.9499	0.935
Economic Value	0.9566	0.9399
Life Sustainability Value	0.9207	0.8909
Biodiversity Value	0.937	0.9219

While the model performance met established thresholds for predictive robustness, certain limitations remain. The SolVES model does not fully account for dynamic user behavior, perceptual biases, or temporal fluctuations in social value expression. Therefore, its application should be complemented by qualitative methods and context-specific analyses to support more comprehensive and adaptive decision-making processes.

### 3.5. Comparison of SolVES model evaluation and social media perception data

By integrating the evaluation results from the Social Values for Ecosystem Services (SolVES) model with social media data and applying social network analysis, this study identified key elements contributing to the multidimensional social values of Luogang Park in Hefei. (1) Aesthetic value: The term “Yuanboyuan” functioned as a central node, frequently co-occurring with locations such as “Baihuayuan” and “Yueji”, forming the core spatial cluster associated with visual and landscape appreciation. (2) Economic value: “Yuanbo Town” exhibited strong associations with hotel and catering-related terms, indicating its central role in supporting tourism-driven economic functions within the park. (3) Recreational value: Terms related to “check-in” “photography” and “kite flying” were highly active in the network, highlighting behavior patterns aligned with social sharing and leisure engagement. (4) Life-sustaining value: Although references to “comfort” “air quality” and “scenery” were present, the frequency and connectivity of these terms remained low, indicating limited public engagement with the park’s ecological and restorative attributes.

**Fig 10:**
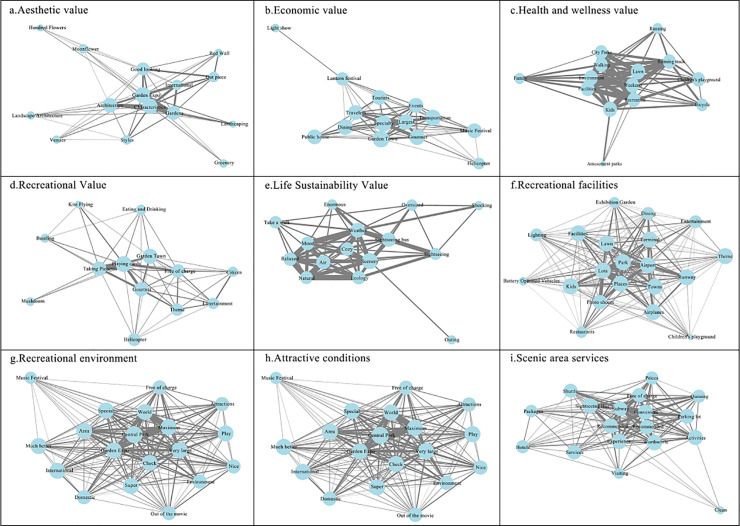
Comparative Validation of Perceptual Evaluation Based on Social Media Data.

Further network analysis of social media discourse-focused on recreational facilities, environmental quality, spatial attractiveness, and service experience-mapped pathways to enhance social value perception and spatial responsiveness. The results emphasize the importance of optimizing spatial layout and communicative design elements to align ecological value delivery with user expectations. Considering the park’s role in the broader urban ecological network, future planning should prioritize the reinforcement of ecosystem integrity and resilience to support sustainable and perceptually inclusive management strategies (Fig 10).

## 4. Discussion

### 4.1. Drivers and differentiation of social value patterns

The pronounced spatial polarization of social values in Hefei Luogang Park challenges the assumption of spatially homogeneous multifunctionality in MSG planning [58]. In this park, aesthetic and recreational values cluster in cultural zones, while ecological values remain dispersed and underrecognized. [58]. We show that multifunctionality is in fact a perceptually and spatially segregated outcome, mediated not only by physical design but also by what we term “perceptual accessibility” which integrates physical proximity with the cognitive salience of landscape features. This concept extends environmental affordance theory by demonstrating that a landscape’s functional potential is co-shaped by socio-cultural narratives and historical legacies guiding public valuation [59].

Cultural landmarks such as the former airport terminal further illustrate how historical memory is spatially encoded and symbolically reactivated in post-industrial landscapes, advancing Cultural Ecosystem Services (CES) theory beyond static valuation [60]. The three-tier agglomeration structure reflects a systematic relationship between functional zoning and social value perception.[14] In high-intensity cultural and recreational zones, aesthetic and recreational value concentration is reinforced by deliberate design choices-visual landmarks, accessible pathways, and clustered amenities-that lower the perceptual threshold for value recognition and encourage repeated engagement, consistent with environmental affordance theory [61,62].

Conversely, the dispersed distribution of ecological values in conservation zones reflects a structural mismatch between ecological function and public perception.These areas are not ecologically deficient but lack the interpretive and infrastructural conditions that render ecological value legible to general visitors-a gap that may perpetuate a self-reinforcing cycle of low visitation, reduced recognition, and diminished management priority. Targeted interventions such as interpretive trails, ecological signage, and digital way finding could extend perceived accessibility into these underrecognized zones [63,64].

### 4.2. Spatial optimization and governance strategies

Building on these insights, we propose a multidimensional optimization framework. Cultural-recreational corridors and nature education zones serve not only as physical interventions but also as mechanisms to reconnect fragmented socio-ecological values [65–67]. The integration of intelligent technologies (e.g., VR/AR) offers novel ways to enhance experiential learning and emotional engagement, particularly among younger demographics [68,69]. The proposed “super IP” model further provides a replicable approach to monetize cultural assets while preserving historical narratives, bridging economic sustainability with heritage conservation [70].

From a governance perspective, public-private partnerships and community-driven management reflect a shift toward polycentric and adaptive governance, essential for long-term sustainability in complex urban systems [71,72]. These strategies collectively advance a social-ecological systems approach that aligns spatial design with human behavior and institutional capacity.

### 4.3. Limitations and future directions

This study has several limitations. The social media data, limited to the park’s inaugural year, may not capture evolving public perceptions.Future research should incorporate longitudinal tracking to assess value dynamics across developmental stages. Additionally, the exclusion of microclimatic and acoustic variables limits the comprehensiveness of environmental drivers; future models should integrate multi-sensory and micro-environmental data to better explain perceptual variability [73,74].

Methodologically, while SolVES demonstrated high predictive accuracy, its reliance on static environmental layers constrains its capacity to capture temporal and behavioral fluidity. Coupling SolVES with agent-based modeling or real-time mobility data could enhance dynamic assessment. Finally, expanding social media sources to include platforms with diverse user demographics (e.g., Weibo, Douyin) would improve the representativeness of perceptual data and mitigate platform-specific biases [75,76].

### 4.4. Practical and policy implications

This study provides actionable insights tailored to key stakeholders involved in the governance of large-scale green spaces. Urban planners and landscape architects can utilize the spatial patterns of social values obtained from the SolVES-social media framework. These patterns can inform the design of multifunctional zones—such as cultural-recreation corridors and nature education areas—that harmonize ecological restoration with public perception [77]. Park managers and administrators can enhance place attachment and respond effectively to visitor needs by implementing participatory tools like perception surveys and social media monitoring, thereby enabling adaptive management and inclusive programming that incorporates heritage interpretation [52,78]. Policymakers and local governments are encouraged to integrate perception-driven social value assessment into urban revitalization and green-space governance frameworks. This integration can promote equitable access, reinforce community identity, and facilitate a transition from a supply-oriented model to one that is both ecologically sustainable and socially responsive [14,22]. This integrated approach collectively supports evidence-based, human-centered planning for expansive urban green spaces in post-industrial settings [61].

## 5. Conclusion

This study established and validated an integrated “spatial quantification–semantic perception” framework to assess social values of cultural ecosystem services in Hefei Luogang Park, a representative mega-scale green space regenerated from brownfield. By coupling SolVES modeling with social media analytics, we identified pronounced spatial polarization: aesthetic and recreational values formed distinct hot spots in culturally programmed zones, while ecological values remained significantly underrecognized in peripheral natural areas. This pattern reveals a perceptional disconnect influenced by functional zoning, accessibility constraints, and historical legacy.

The research provides three fundamental contributions. Theoretically, it advances cultural ecosystem service assessment by incorporating historical memory and environmental justice dimensions, demonstrating how social values are co-produced in post-industrial landscapes. Methodologically, it pioneers the integration of spatial modeling with semantic analysis in brownfield regeneration contexts, offering a transferable framework that effectively bridges quantitative cartography with qualitative public sentiment. Practically, the study delivers actionable strategies—including cultural-recreational corridors, nature education zones, and IP-based governance models—that collectively support the development of equitable, multifunctional green spaces aligned with contemporary planning initiatives.

While this framework represents a significant advance, future research should incorporate longitudinal perceptual monitoring and multi-sensory environmental data to capture more dynamic value formations. By continuing to refine the synergy between spatial science and social perception, this approach provides a robust foundation for planning sustainable, inclusive, and resilient urban green spaces in an era of rapid urbanization.

## Supporting information

S1 Table Survey checklist for visitor demographic analysis. (XLSX)

S2 Table GIS data layers for environmental elements and social value density analysis. (XLSX)

S3 Table Data for social value distribution and factor relationship analysis. (XLSX)

S4 Table Geo-environmental contribution data for social value types. (XLSX)

S5 Table Social media perception evaluation dataset. (XLSX)
